# Subacute Right Ventricle Perforation by Pacemaker Lead Presenting with Left Hemothorax and Shock

**DOI:** 10.1155/2015/983930

**Published:** 2015-02-18

**Authors:** Julianne Nichols, Natalie Berger, Praveen Joseph, Debapriya Datta

**Affiliations:** ^1^Department of Medicine, University of CT Health Center, 263 Farmington Avenue, Farmington, CT 06030, USA; ^2^Division of Pulmonary and Critical Care Medicine, University of CT Health Center, Farmington, CT 06030, USA

## Abstract

Cardiac perforation by pacemaker is a rare but potentially fatal complication. Acute perforations occurring within twenty-four hours of insertion of pacemaker can lead to hemopericardium, cardiac tamponade, and death. Hemothorax occurring as an acute complication of pacemaker insertion is reported but extremely rare. Previously, hemothorax and shock as a subacute complication following pacemaker insertion have not been reported. We report the case of an 85-year-old patient who presented with shock from hemothorax caused by pacemaker perforation, two weeks after insertion. Device interrogation showed normal function. Chest X-ray and echocardiogram missed lead dislocation and the diagnosis was made on computed tomogram (CT) of the chest. Following surgical repair, a new ventricular pacemaker was placed transvenously in the right ventricular septum. This case illustrates that CT scan of the chest should be performed in all patients in whom cardiac perforation by pacemaker is suspected but not diagnosed on chest X-ray and echocardiogram. Normal functioning of pacemaker on device interrogation does not exclude perforation.

## 1. Introduction

Cardiac perforation by pacemaker is a rare but potentially fatal complication. Acute perforations occurring within twenty-four hours of insertion of pacemaker can lead to hemopericardium, cardiac tamponade, and death. Hemothorax as an acute complication of pacemaker is extremely rare and has not been reported as a subacute complication. We report the case of an 85-year-old patient with shock from hemothorax caused by pacemaker perforation, two weeks after insertion, which was missed on chest X-ray and echocardiogram but diagnosed on computed tomogram (CT) of chest.

## 2. Case Description

**Figure 1:**
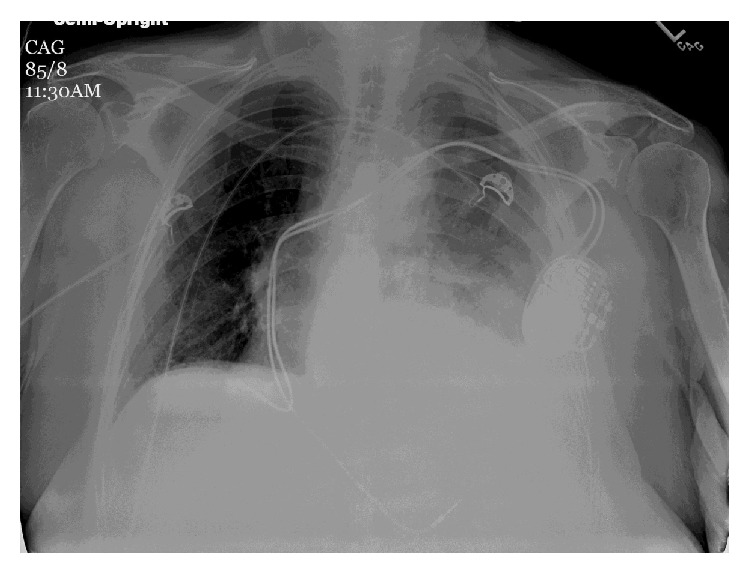
Chest X-ray AP view: opacity at left base consistent with a moderate left-sided pleural effusion; pacemaker leads appear to be present in the right ventricle.

**Figure 2:**
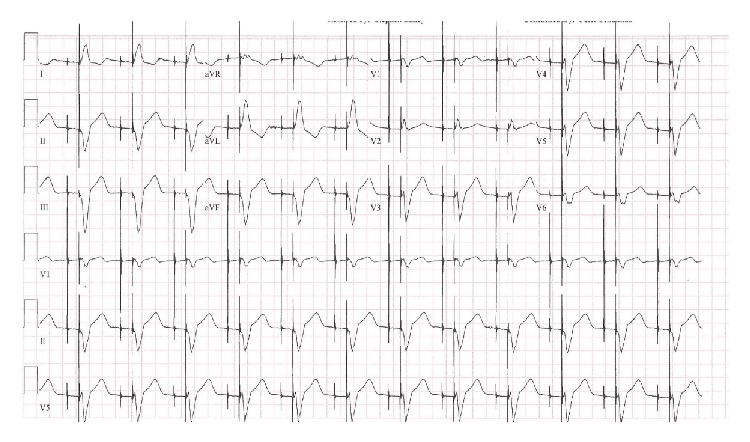
EKG showing AV sequential pacing.

An eighty-five-year-old female presented to the emergency room with complaints of sudden onset substernal chest pain and dizziness. Her past medical history was significant for atrial fibrillation. She had a pacemaker inserted at another facility 2 weeks before. No perioperative complications were reported and patient had been doing well without any complaints till the day of presentation. Medications included dabigatran for her atrial fibrillation. On examination, the patient was diaphoretic but in no respiratory distress. Temperature was 97°F, pulse 96/minute, irregular; systolic blood pressure was 70 mm Hg; respiratory rate was 22/minute; and oxygen saturation was 95% on room air. No jugular venous distension was noted. Cardiac examination revealed irregular rhythm, no murmur or rub. Lung auscultation revealed slightly diminished breath sounds at left base. Chest X-ray showed a mild-to-moderate left-sided pleural effusion; pacemaker leads appeared to be in the right ventricle (Figure 1). Electrocardiogram (EKG) showed trioventricular (AV) sequential pacing (Figure 2). Echocardiogram (ECHO) revealed no wall motion abnormality with normal ejection fraction. Pacemaker leads were visualized in the right ventricle (RV) on ECHO. Her hemoglobin was 8 gm%; prothrombin time (PT) was 19 seconds; and INR was 2.9.

**Figure 3:**
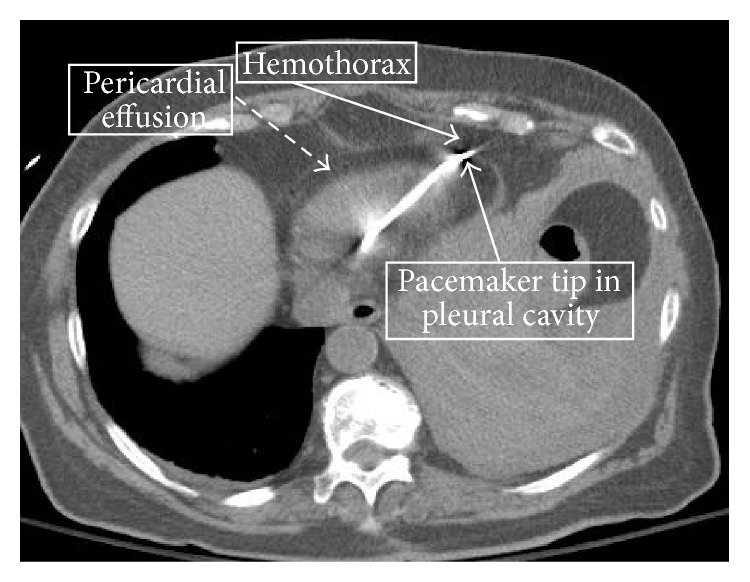
CT scan of chest showing the pacemaker wire extending to the left pleural cavity with a moderate pleural effusion and a small localized adjoining pericardial effusion.

No pacing or sensing failure was noted on device interrogation. The patient's hypotension persisted despite vigorous intravenous fluid resuscitation including transfusion of 2 units of blood cell and 6 units of fresh frozen plasma and improvement in her hemoglobin to 11 gm%. A computed tomogram (CT) of the chest was performed which showed a moderate left pleural effusion, with the pacemaker wire extending to the left pleural cavity and a small localized pericardial effusion adjoining the pleural effusion (Figure 3). The patient's hemodynamic instability was not due to tamponade effect as the pericardial effusion was small but due to continued hemorrhage into the pleural cavity due to her coagulopathic state from being on dabigatran for her atrial fibrillation, which was not fully corrected by fresh frozen plasma infusion.

**Figure 4:**
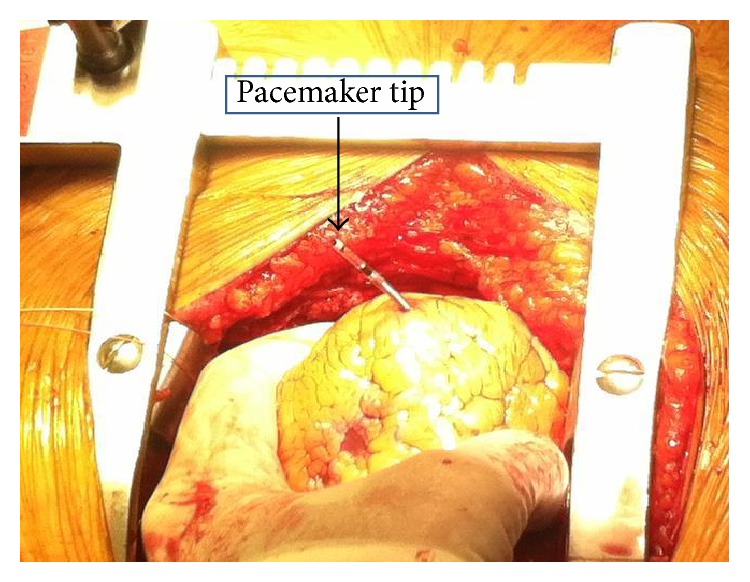
The tip of pacemaker lead is seen protruding out of the right ventricle.

The patient was taken to the operating room where she underwent a median sternotomy. The pacemaker lead was found to be protruding out of the right ventricle (Figure 4). The external portion was cut with the remaining intracardiac portion of the pacemaker lead left in place. The perforation of the RV wall was repaired. The hemothorax and hemopericardium were evacuated. Subsequently, the patient's hemodynamic status improved. Two days later, the remaining portion of the pacemaker lead was removed transvenously. A new pacemaker was inserted transvenously and placed on the right ventricular septum. The patient did not have further complications and was discharged home subsequently. On follow-up 6 weeks later, she was doing well.

## 3. Discussion

Though rare, cardiac perforation in patients after pacemaker implantation is potentially life-threatening. Reported rate of occurrence of pacemaker perforation is 0.1–3% [1–4]. Perforations occurring within 24 h after implantation are labeled as acute; those occurring within one month after implantation are subacute while perforations which occur after one month are labeled as chronic [5, 6]. Pacemaker perforations may occur through the walls of the large veins, atria, or ventricles. Perforations involve the RV apex, which is thinner, more commonly than the intraventricular septum or the outflow tract [6].

The pathophysiology behind the occurrence of pacemaker perforation is not clearly understood but is believed to be multifactorial and related to the pacemaker lead dimension and overtorquing of the leads [2]. It is hypothesized that increased pressure force exerted by the thin pacemaker leads per unit of the ventricular wall, as well as the imbalance between the pacemaker lead tip forces and the ventricle, result in perforation [5, 7].

Symptoms and signs of pacemaker perforation depend on the location of the displaced lead [3, 5]. The pacemaker may perforate the vascular chamber and migrate to the pericardial cavity, pleural cavity, mediastinum, lung, diaphragm, chest muscles, and the peritoneal cavity [6]. The common symptoms are chest pain, dyspnea, abdominal pain, and dizziness [8]. Hiccups due to diaphragm contraction or visible chest muscle contraction may occur from displaced pacemaker stimulation [3, 9]. Hemodynamic instability may occur if hemopericardium develops and leads to cardiac tamponade which can result in shock, heart failure, and cardiac arrest [6]. Occurrence of left hemothorax with pacemaker perforation is very rare and can also result in hemodynamic instability which may be life-threatening, as in this patient. Perforated pacemaker leads can result in pacing and sensing failures [8, 10]. Changes of pacing parameters such as capture threshold and sensing threshold depend on the location of the displaced lead tip. Loss of consciousness, heart failure, and cardiac arrest may occur because of pacing failure [2, 8].

Chest X-ray is an easy and commonly used diagnostic method for detecting pacemaker perforation. On chest X-ray, a diagnosis of pacemaker perforation can be made if the lead is located beyond the confines of the cardiac silhouette. A lateral view of the chest should always be performed as it can localize the position of the pacemaker lead more accurately. Chest X-ray can also detect extracardiac complications such as pleural or pericardial effusion and pneumothorax. Echocardiography (ECHO), a simple and noninvasive test that can be performed easily at the bedside, can also help to assess electrode location and detect presence of the pacemaker lead tip in the pericardium and presence of pericardial effusion. However both of these diagnostic tests have their limitations and the location of the pacemaker lead tip may not be correctly located, as is evident in this case. CT scan of the chest is currently regarded as the gold standard in the diagnosis of pacemaker lead perforations [11, 12]. Performing CT scans is standard care in most departments dealing with cardiac implantable electronic devices implants. Chest CT accurately reveals pacemaker lead displacement which can sometimes be missed by chest X-ray or ECHO as in this patient. In addition, it can confirm the presence of an associated pericardial effusion/hemopericardium or pleural effusion/hemothorax [13]. However, it should be borne in mind that the position of pacemaker wires may be misinterpreted on CT due to artifacts. An atypical position of the pacemaker lead with a left-sided pleural effusion, decreased hemoglobin, and hemodynamic instability should lead to the diagnosis without the need for a CT scan.

Pacemaker interrogation should be performed as part of the evaluation in patients with suspected pacemaker perforation. However, normal function and absence of sensing and pacing failure do not rule out pacemaker perforation [2]. Several studies have reported various factors that serve as predictors of lead perforation. These include temporary leads, steroid use, active fixation leads, low body mass index (<20 kg/m^2^), older age, female gender, and concomitant anticoagulation [14, 15]. In this case, three of these predictor factors were present: older age, female gender, and ongoing anticoagulation. The only known protective factor for cardiac perforation is right ventricular systolic pressure >35 mm Hg, which is attributed to coexisting right ventricular hypertrophy [15].

Management depends on patients' hemodynamic status, patients' symptoms, and the presence of associated pericardial or pleural effusion [16]. Emergent surgical management is required if the patient is hemodynamically unstable or if the patient has a large pericardial effusion where tamponade may be imminent or a large pleural effusion with respiratory impairment is present or imminent [16, 17]. In such cases, sternotomy with surgical removal of the perforating leads, evacuation of effusions, and repair of tear should be performed. In cases of lead perforation outside the pericardium, as in this patient, cardiac surgery or video-assisted thoracoscopic surgery is recommended for cutting the extracardiac portion of the tip, repairing the tear and then removing the remaining intracardiac portion of the lead removed transvenously [6, 8]. In hemodynamically stable patients, the pacemaker can be extracted by direct traction or percutaneous lead extraction in the operating room, under close echocardiographic or fluoroscopic monitoring, with surgical backup being available [5, 18]. Lead extraction should be followed by new lead placement in a different location, preferably in the right ventricular outflow tract or the intraventricular septum.

No consensus exists regarding the appropriate management of lead perforation in stable patients without symptoms or the management of chronic lead perforation without pacemaker malfunction. Some experts recommend lead removal in all such cases [12] while others [2] recommend against the removal of a chronically perforated lead without pacemaker malfunction.

## 4. Conclusion

At the present time, pacemaker insertion is a commonly performed therapeutic intervention for the management of specific arrhythmias. Though the complications arising from pacemaker insertion are uncommon, they can be life-threatening and hence should be considered in all patients with cardiac pacemakers in the appropriate clinical setting. Normal function on device interrogation does not rule out perforation. Chest X-rays and echocardiogram, though easy to perform, may miss displaced pacemaker leads. CT chest is the gold standard for the diagnosis of pacemaker lead perforation. Hence, CT scan of the chest should be performed in all patients in whom pacemaker perforation is suspected but not diagnosed on chest X-ray and ECHO.
